# Protocol for a mixed methods process evaluation of a hybrid implementation-effectiveness trial of a scaled-up whole-school physical activity program for adolescents: Physical Activity 4 Everyone (PA4E1)

**DOI:** 10.1186/s13063-020-4187-5

**Published:** 2020-03-17

**Authors:** Matthew Mclaughlin, Jed Duff, Rachel Sutherland, Elizabeth Campbell, Luke Wolfenden, John Wiggers

**Affiliations:** 1 Hunter New England Population Health, Longworth Avenue, Wallsend, 2287 NSW Australia; 2 School of Medicine and Public Health, University of Newcastle, University Dr, Callaghan, NSW 2308 Australia; 3 Hunter Medical Research Institute, Lot 1, Kookaburra Cct, New Lambton Heights, 2305 NSW Australia; 4 Priority Research Centre for Health Behaviour, University of Newcastle, Callaghan, 2308 NSW Australia

**Keywords:** Process evaluation, Implementation, Physical activity, Adolescents, Schools, Mixed methods, Randomised controlled trial

## Abstract

**Background:**

Physical Activity 4 Everyone (PA4E1) is a physical activity program for secondary schools located in low-socioeconomic areas. Over a 24-month period, schools in the program arm of a cluster randomised controlled trial (n = up to 38 schools) will receive a multi-component implementation support strategy to embed the seven school physical activity practices of the PA4E1 program. This article describes the process evaluation of the PA4E1 hybrid implementation-effectiveness trial. The process evaluation aims to describe the fidelity and reach of the implementation support strategies using quantitative data; and to describe the acceptability, appropriateness and feasibility of the implementation support strategies and physical activity practices to school stakeholders using mixed methods.

**Methods:**

Quantitative and qualitative data will be collected from participants (Physical Education teachers, in-School Champions, students) in the program arm. Data collection will involve semi-structured interviews, focus groups, a fidelity monitoring log, a fidelity checklist, surveys, and routinely collected administrative and website data. Quantitative data will be analysed descriptively and qualitative data will be analysed thematically within and across data sets. Triangulation between data sources will be used to synthesise findings regarding the implementation and potential mechanisms of impact of PA4E1 on school physical activity practice adoption, with respect to context.

**Discussion:**

Results of the process evaluation will facilitate the interpretation of the findings of the trial outcomes. It will comprehensively describe what was actually implemented and identify the potential contribution of the various components of the implementation support strategy to the school physical activity practice adoption outcomes. Findings will inform future improvement and scale-up of PA4E1 and approaches to implementing secondary school-based physical activity programs more broadly.

**Trial registration:**

Australian New Zealand Clinical Trials Registry ACTRN12617000681358 registered 12 May 2017.

## Background

The health and economic burden of physical inactivity is well established [1, 2]. The global prevalence of physical inactivity during adolescence is high, with 80% of adolescents self-reporting less than the guideline of 60 minutes moderate-vigorous intensity physical activity daily [3, 4]. Additionally, adolescent physical activity habits track into adulthood [5]. As such, adolescence is considered a critical period in the establishment of physical activity behaviour.

School-based programs are recommended as key settings to provide opportunities for physical activity [6]. Secondary schools provide access to adolescents and their families for ongoing periods in a critical development phase. In addition, schools can have the resources, professional skills and policies required to encourage physically active lifestyles [7, 8]. Whilst secondary schools are a key setting for physical activity promotion, it should be acknowledged that school leaders may not identify physical activity promotion as an explicit priority [9, 10]. Despite the potential of implementing physical activity programs in this setting, few high-quality programs have targeted secondary schools, with systematic reviews suggesting programs are typically ineffective in improving moderate-vigorous intensity physical activity [11, 12].

A systematic review suggested that there may be a positive relationship between the level of implementation of school-based physical activity programs (fidelity, dose received/delivered, quality) and health outcomes [13]. The lack of effectiveness of many school-based programs has, in part, been attributed to poor implementation of the program components [11, 13]. As a consequence, greater focus is being placed on applying implementation science methods to ensure trial programs are delivered with sufficient fidelity, dose and quality to enhance student physical activity [14]. To accelerate research translation, hybrid implementation-effectiveness trials are now recommended, whereby researchers simultaneously develop and test the effects of programs on health outcomes, as well as the impact of strategies to support and improve implementation of the program components [15].

In addition to outcome evaluation, process evaluation of implementation trials are recommended to contextualise the primary findings of such trials, and to help understand ‘*what did (and did not) work, and why*?’ [16]. The UK Medical Research Council’s (MRC) framework for designing and evaluating complex interventions (programs) recommends process evaluations be undertaken as part of all randomised trials; as they describe important contextual factors, and can clarify causal mechanisms to aid interpretation of trial findings and explain variations in trial outcomes [16]. However, a recent systematic review of randomised controlled trials (RCTs) of school-based physical activity programs highlighted that process evaluations were rare, undertaken in just four of the 17 trials identified [11, 17–20]. Furthermore, none of the process evaluations described or assessed the delivery of implementation support strategies to facilitate implementation of the program [17–20], nor were process evaluation methods specified in a publically available protocol or register [17–20]. Without robust process evaluations to understand what and how well implementation support strategies were delivered as part of a school physical activity trial, there is little basis to suggest how implementation could be improved, which is important in guiding decisions on whether programs should be delivered at scale to maximise population health impact.

The objective of the study is to conduct a comprehensive process evaluation as part of a cluster RCT of a secondary school-based physical activity program ‘Physical Activity 4 Everyone’ (PA4E1) [21]. The hybrid implementation-effectiveness trial seeks to evaluate the effectiveness of a multi-component implementation support strategy to improve implementation, at scale, of the seven physical activity practices of the PA4E1 program (primary outcome), and also assesses the effect on student physical activity (secondary outcome); a separate protocol is available for the implementation-effectiveness outcomes [21]. The aims of the process evaluation are to:

1. To describe fidelity and adaptations to the PA4E1 program (implementation support strategies and physical activity practices).
2. To describe the reach of the PA4E1 implementation support strategies within schools.
3. To describe acceptability, appropriateness and feasibility of the PA4E1 implementation support strategies from the perspective of Physical Education (PE) teachers and in-School Champions.
4. To describe acceptability, appropriateness and feasibility of the PA4E1 physical activity practices from the perspective of PE teachers, in-School Champions and students.

### The PA4E1 Program

The PA4E1 program is a multi-component secondary school physical activity program which targets adolescents (aged 12–17 years) from secondary schools located in low-socioeconomic areas [21]. Briefly, PA4E1 is based on the Health Promoting Schools framework; taking a whole-of-school approach, which includes delivery of seven physical activity practices that span the school curriculum, school environment and partnerships and services levels [21, 22]. The design of the program was guided by social cognitive [23] and social-ecological theory [24]. An initial 2-year efficacy trial (RCT design) of PA4E1 [25] conducted from 2012 to 2014 in ten schools (five program: five control) in New South Wales, Australia, found positive significant effects on student total-day moderate-vigorous intensity physical activity [26], reductions in weight gain [27], as well as being cost-effective [28]. Schools were offered six implementation support strategies to help them adopt the seven physical activity practices over 24 months, with four of the five program schools adopting all practices and the remaining school adopting six practices [26]. A RCT of the scale-up of the PA4E1 program will be undertaken, employing a hybrid implementation-effectiveness RCT design in up to 76 schools (38 program: 38 control) across four local health districts in NSW, Australia [21].

**Fig. 1:**
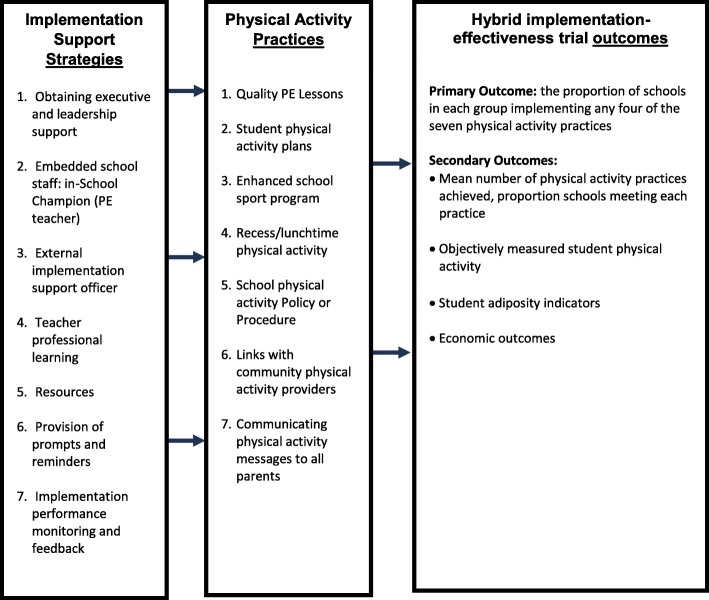
PA4E1 program logic model: Inclusive of implementation support strategies, physical activity practices and evaluation outcomes of the hybrid implementation-effectiveness evaluation. For detailed descriptions of the implementation support strategies and the physical activity practices see Additional file 2

The PA4E1 program aims to embed the same seven evidence-based physical activity practices that have been retained from the efficacy trial within the school environment (referred to as physical activity ‘practices’) [21, 25]. The implementation support strategy consists of seven implementation support strategies, which are behaviour change techniques designed to overcome the barriers to embedding the physical activity practices into the school (referred to as *strategies).* The physical activity practices and implementation support strategies are listed in Fig. 1 and detailed in Additional file 2 [21].

When referring to the ‘PA4E1 program’ we include both the physical activity ‘practices’ and the implementation support ‘strategies’ [21]. Figure 1 shows the PA4E1 program logic model, inclusive of implementation support *strategies* (the implementation support strategy), physical activity *practices* (the physical activity program) and the hybrid implementation-effectiveness evaluation outcomes.

The implementation support strategies were adapted from those used in the original efficacy trial to enable feasible delivery at scale, including modification to their mode of delivery [21, 25]. A summary of the main adaptations from the efficacy trial can be found in the trial protocol [21]. Briefly, two main adaptations to the implementation support strategies were (a) the use of a website to partially replace face-to-face and paper-based delivery modes; (b) the addition of a support strategy, an in-School Champion (an existing Personal Development, Health and Physical Education teacher from within the school, hereafter referred to as PE teachers) to lead the program within schools, supported by an external health promotion Support Officer (employed by the local health district), in substitution of the in-school physical activity consultant (employed by the local health district) used in the original trial [21, 25]. Both these adaptations were to facilitate scale-up and sustainability of the program.

The primary aim of the PA4E1 hybrid implementation-effectiveness evaluation is to assess the impact of the implementation support strategies on school implementation of the physical activity practices over 12 and 24 months, specifically the proportion of schools adopting at least four of the seven physical activity practices [21]. Physical activity practice implementation (primary outcome) will be assessed via Head PE teacher computer-assisted telephone interviews. Secondary outcomes include student physical activity as measured by accelerometer, and student adiposity indicators (a nested study of 30 schools) (Fig. 1) [21].

## Methods

This protocol should be read in conjunction with the protocol for the PA4E1 hybrid implementation-effectiveness RCT [21]. The current process evaluation protocol describes in detail the methodology to be used for a detailed process evaluation. Given the need to provide sufficient and appropriate detail for all such trial evaluation activities, a decision was made to prepare two separate protocols. The trial has been prospectively registered ACTRN12617000681358. Ethical approvals were sought in advance from Hunter New England Human Research Ethics Committee (Ref No. 11/03/16/4.05), University of Newcastle (Ref No. H-2011-0210), NSW Department of Education and Communities (SERAP 2011111), Maitland Newcastle Catholic School Diocese, Broken Bay Catholic School Diocese, Lismore Catholic School Diocese, Armidale Catholic School Diocese, and the Aboriginal Health and Medical Research Council. Digital and hard-copy data will be stored securely at Hunter New England Population Health, Newcastle, Australia.

**Fig. 2:**
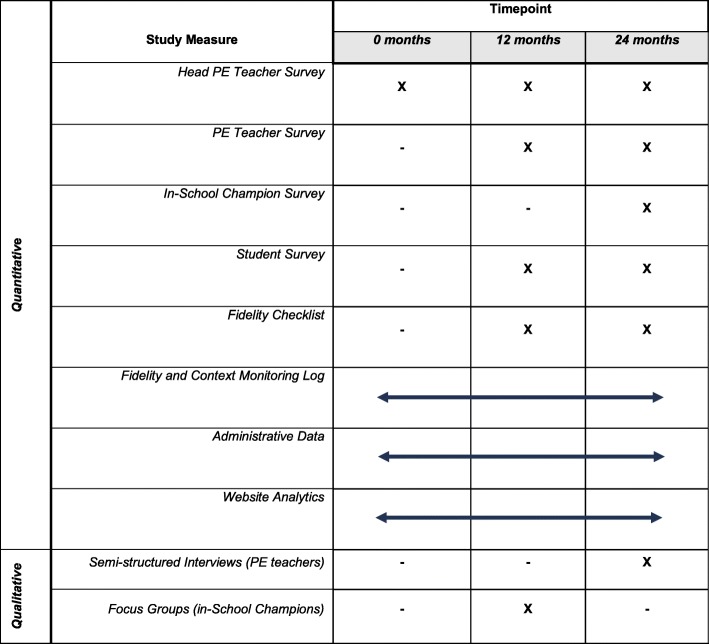
Schedule of PA4E1 process evaluation data collection methods (SPIRIT Figure)

This protocol was developed according to the Standardized Protocol Items: Recommendations for Interventional Trials (SPIRIT) guidelines (Additional file 1 and Fig. 2 [29]). The research will be conducted and reported in accordance with the requirements of the Standards for Reporting Implementation Studies (StaRI) Statement (Additional file 3 [30]). The process evaluation was developed based on the MRC guidance on process evaluations of complex interventions (programs) [16] and incorporates an examination of the three key components of a process evaluation recommended by the MRC – trial context, implementation, and mechanisms of impact [16].

## Data Collection

### Overview of Data Sources

The following measures pertain to the program group only and will not be obtained for the control group. A number of quantitative and qualitative methods will be used to gather insights from school stakeholders pertaining to the implementation of PA4E1. For an outline of these methods and their timing, see Table 1 and Fig. 2.

**Table 1:** Methods for data collection with participants

	Surveys	Semi-structured interviews	Focus groups	Website analytics	Administrative data
Head PE teacher	**✓**				
PE teachers	**✓**	**✓**		**✓**	**✓**
In-School Champions	**✓**		**✓**	**✓**	**✓**
Students	**✓**				

Aims 1–4 will be addressed using the following data sources. Aim 1 will use a fidelity and context monitoring log and a fidelity checklist. To describe reach, aim 2 will use program administrative data, website analytics data and a Head PE teacher computer-assisted telephone survey. To describe acceptability, appropriateness and feasibility, aims 3 and 4 will use data quantitative data from online surveys with PE teachers and in-School Champions, and qualitative data from semi-structured interviews with PE teachers and focus groups with in-School Champions. Aim 4 will additionally use quantitative data from a survey with students. Definitions of key terms used within the process evaluation have been adopted from Proctor et al. [31], see Table 2.

**Table 2:** Definitions of key terms, from Proctor et al. [31]

Key term	Definition
Fidelity	“… the degree to which an intervention was implemented as it was prescribed in the original protocol or as it was intended by the program developers” (p 69)
Reach	“…the integration of a practice within a service setting and its subsystems” (p 70)
Acceptability	“…the perception among implementation stakeholders that a given treatment, service, practice, or innovation is agreeable, palatable, or satisfactory.” (p 67)
Appropriateness	“…the perceived fit, relevance, or compatibility of the innovation or evidence-based practice for a given practice setting, provider, or consumer; and/or perceived fit of the innovation to address a particular issue or problem.” (p 69)
Feasibility	“…the extent to which a new treatment, or an innovation, can be successfully used or carried out within a given agency or setting” (p 69)

### Aim 1

*“To describe fidelity and adaptations to the PA4E1 Program (implementation support strategies and physical activity practices).”*

Two quantitative tools will be employed to address aim 1.

We adopted the conceptual understanding that adaptations may be “…planned or purposeful changes to the design or delivery of an intervention, but they can also include unintentional deviations from the interventions as originally designed” (p. 2, [32]).

#### Fidelity and Context Monitoring Log

Throughout the 24-month program, a monthly meeting of up to 1 hour between the PA4E1 Implementation Team (PA4E1 Management Team and support officers) will be held to continually track adaptations through ‘real-time’ using a modified version of a consistent coding framework of adaptations, FRAME [32–34]. The Implementation Team consists of researchers who developed the program, health service managers, and support officers who are part of the implementation strategy (Fig. 1). This method has previously been found to be feasible to track adaptations [33]. This meeting will be held independent of implementation meetings, to avoid burdening implementation with evaluation measures. Adaptations to implementation support strategies and physical activity practices will be logged using Microsoft Excel. In addition to monitoring fidelity, contextual factors relating to the PA4E1 program (e.g. changes to personnel, policy) will also be discussed and recorded at these meetings.

#### Fidelity Checklist

A study-specific fidelity checklist was developed to assess the dose of the implementation support strategies delivered [31]. The checklist is specific for this study based on the trial protocol’s outline of the seven implementation support strategies [21]. This includes 23 implementation sub-strategies split across the seven implementation support strategies (Additional file 2). Specifically, the checklist will assess the proportion of the seven implementation support strategies (across the 23 sub-strategies) that were implemented, as described in the protocol, to each of the program schools. The checklist will be completed at 12 and 24 months by the PA4E1 Implementation Team.

### Aim 2

*“To describe the reach of the PA4E1 implementation support strategies within schools.”*

Three quantitative tools will be used to address aim 2. In addition, the three tools will be used to create a reach checklist, which will be developed to assess the proportion of schools obtaining each of the 23 implementation support sub-strategies (across the seven implementation support strategies) by the PA4E1 Implementation Team (Additional file 2).

#### Administrative Data

A collation of monitoring documents will be used routinely during the implementation of the trial by the PA4E1 Implementation Team to monitor the reach of implementation support strategies. These include logging the number of contacts with in-School Champions, in-School Champion training attendance, schools receiving resources and schools accessing funding for their in-School Champion. Logs will be updated throughout the program 24-month program in Microsoft Excel.

#### Website Analytics

To assess the proportion of PE teachers and in-School Champions accessing digital implementation support strategy components, website analytics will be taken from Google Analytics. Specific analytics will include the number of pages viewed and time on site.

#### Head PE Teacher Survey

Head PE teachers will be asked to participate in a computer-assisted telephone interview survey with trained research staff at 12 and 24 months. Where data is not available to assess reach using objective measurement from administrative data and website analytics data, questions will be asked of the Head PE teacher at each school. Questions will assess whether schools implemented certain components of the implementation support strategies (e.g. obtained executive support and leadership through forming a committee).

### Aim 3 and Aim 4

Aim 3: “*To describe acceptability, appropriateness and feasibility of the PA4E1 implementation support strategies from the perspective of PE teachers and in-School Champions*.”

Aim 4: “*To describe, acceptability, appropriateness and feasibility of the PA4E1 physical activity practices from the perspective of PE teachers, in-School Champions, and students*.”

To address aims 3 and 4, mixed methods will be used. Specifically, four quantitative tools and two qualitative tools will be used to address the aims, in addition to the quantitative tools used in aims 1 and 2 described above.

### Quantitative Data

#### Head PE Teacher Survey

Computer-assisted telephone interviews administered by trained interviewers with Head PE teachers will measure acceptability, appropriateness and feasibility of the PA4E1 program overall at 12 and 24 months using three 4-item valid and reliable scales developed by Weiner et al., on a 5-point Likert scale: the Acceptability of Intervention Measure (AIM), Intervention Appropriateness Measure (IAM) and the Feasibility of Intervention Measure (FIM) [35]. The authors have used computer-assisted telephone interviews previously with similar studies and found high (76–82%) response rates [36].

#### PE Teacher Survey

PE teachers will be emailed and asked to complete an online survey at 12 and 24 months via a link. This survey will collect data from the perspective of PE teachers regarding acceptability, appropriateness and feasibility of the PA4E1 program overall, using the same tools as for Head PE teachers above [35]. To ensure high participation rates, in-School Champions will be encouraged to prompt PE teachers to complete these surveys.

#### In-School Champion Survey

In-School Champions will be asked by email to complete an online survey at 24 months. This survey will ask about the acceptability, appropriateness and feasibility of each of the seven physical activity practices and each of the seven implementation support strategies within PA4E1, using reduced item adapted versions of the AIM, IAM and FIM tools [35]. Specifically, the single items with the highest loadings in previous confirmatory factor analysis will be asked on a 5-point Likert scale. Therefore for IAM and FIM, the questions “I think it’s suitable for our school” and “I think it’s doable for our school” will be asked [35]. With the exception of the AIM tool where the question “I like it” will be used, as items with higher loadings within this construct were considered to be not easily understood for the participant group in the study context [35]. To ensure high participation rates in this survey, in-School Champions will be prompted by their Support Officer to complete the survey.

#### Student Surveys

Students will be asked to complete a survey using a tablet during routine trial outcome data collection at 12 and 24 months. This survey will collect data from the perspective of students regarding acceptability and appropriateness of three PA4E1 physical activity practices relating to the students (Student Physical Activity Plans, Recess and Lunchtime Activities and Community Links respectively [21]). The survey will also collect sociodemographic characteristics of participants (e.g. age, sex, language spoken). In the absence of valid and reliable questions, study-specific questions were developed specific to the PA4E1 physical activity practices: Student Physical Activity Plans, Recess and Lunchtime Activities and Community Links [21]. Specifically, for each practice, students will be initially asked: “did you take part in the following?” (reach), and those who reported participating will be asked: “did you enjoy it?” (acceptability), and “did it help make you more active?” (appropriateness), on a binary scale (yes, no).

### Qualitative Data

Qualitative data will be collected via semi-structured interviews with PE teachers and focus groups with in-School Champions. All observations and interviews will be carried out by the same student researcher (MM), under the guidance of a trained mixed methods researcher (JD).

#### Semi-structured Interviews | PE Teachers

A purposeful sub-sample of PE teachers (up to *n* = 18 PE teachers, *n* = 6 schools) will be invited to take part in interviews conducted face to face at their respective schools. Schools will be sampled across all four local health districts involved in the program. Both high- and low-implementing schools will be sampled, based on physical activity practice implementation data obtained from in-School Champions through the program website after 12 months of implementation support. To ensure high participation rates, recruitment to interviews will be through contact between in-School Champions and support officers. Interviews will be semi-structured, using a flexible interview guide, which will be adapted as necessary after a few interviews have taken place. The PE teachers will be asked about their experiences of the program, including both the implementation support provided and the physical activity practices. Interviews will be conducted within the second year of implementation support.

#### Focus Groups | in-School Champions

Interactive face-to-face focus groups with in-School Champions will be conducted after 12 months of the program. In-School Champions from all 24 schools will be invited to participate. To ensure high participation rates, focus groups will be organised on the same day, and at the same venue, as an implementation training workshop for in-School Champions. In-School Champions will be asked broad questions relating to the program (both physical activity practices and implementation support strategies) using a semi-structured guide. Topics will include what did and did not work, and the suitability of the program for scale-up with regard to physical activity practices and implementation support strategies. Whilst an interview guide will be used, participants will be encouraged to discuss additional issues.

### Data Analysis

Aims 1 and 2 will be analysed descriptively using quantitative data. To address aims 3 and 4, a mixed methods approach will be utilised. Researchers responsible for the process evaluation will be aware of the effect evaluation outcomes (implementation and effectiveness) as they emerge, and vice versa.

### Quantitative Data Analysis

Survey data from Head PE teachers, PE teachers, in-School Champions and students will be analysed producing descriptive statistics. Adaptations to the PA4E1 program will be reported descriptively, with adaptations summarised using a modified version of a consistent coding framework of adaptations, FRAME [32, 34]. Fidelity will be reported as the number of schools provided each of the 23 implementation support sub-strategies (Additional file 2) and as an overall fidelity score across schools. The overall fidelity score will be calculated based on the number of implementation support sub-strategies provided or offered to schools. Reach will be reported descriptively as the proportion of schools up taking each of the implementation support sub-strategies (Additional file 2) and as an overall reach summary score across schools. A reach score for each school will be calculated based on the number of implementation support sub-strategies taken up by schools.

### Qualitative Data Analysis

All focus group and interviews will be audio-recorded and transcribed verbatim. The transcripts will be checked for accuracy, with corrections made as appropriate. Identifiable comments will be anonymised prior to importing into NVivo. All qualitative data will be thematically analysed using QSR NVivo [37].

For the analysis of focus group and interview data, a commonly utilised six-phase method [38] will be used. Two researchers will independently code the same subset of transcripts (10%), familiarising and reacquainting them with the data, respectively. This early coding will be inductive, but as themes are forming the authors will refer to Proctor et al. to help organise the findings [31]. As such, both researchers (MM, JD) will independently develop their own inductive coding scheme. Codes will be discussed by the two coders, and coding schemes will be refined and amended via an iterative process prior to the lead researcher (MM) continuing further coding of the transcripts.

Members of the research will discuss interim themes and reach consensus. Initially, focus group and interview data will be analysed separately. Similar coding schemes will be used for the qualitative data collected via interview and focus groups, but where one set of results uncovers a theme not covered by other results, additions may be made.

As the interview and focus group data sets come from PE teachers and in-School Champions, respectively, they will be comparatively triangulated to address completeness, convergence, and dissonance of key themes.

### Mixed Methods Analysis

Mixed methods studies mix the data from quantitative and qualitative data sets, and this is dependant on: (a) level of integration, (b) priority of quantitative and qualitative strands, (c) timing of data collection, (d) where and how the quantitative and qualitative strands will be combined [39]. How this will occur is described below.

**Fig. 3:**
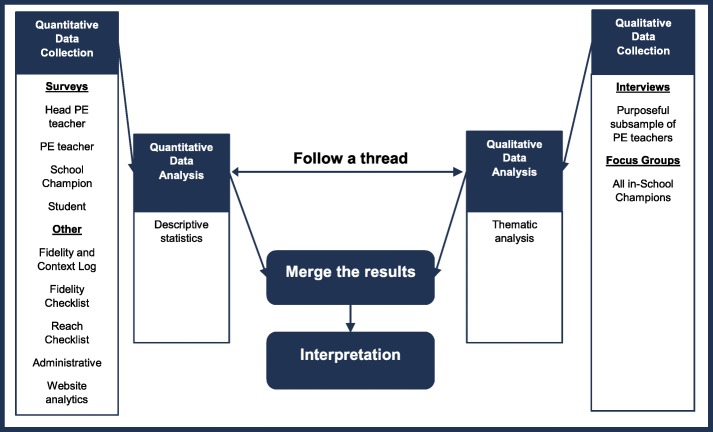
Analytical procedure for the PA4E1 mixed methods process evaluation

Qualitative and quantitative data will be collected throughout the study. Mixing of the qualitative and quantitative data sets will occur during analysis, with both quantitative and qualitative data strands being given equal emphasis as they both address aims 3 and 4 equally. Analysis of the quantitative data to produce descriptive statistics will occur concurrently with generating the initial codes of the qualitative data. A diagram of the process evaluation analytical procedure is provided in Fig. 3.

An approach called ‘following a thread’ will be employed, whereby key themes that arise in one data set will be further explored or explained with another [40]. For example, quantitative data might suggest low acceptability of the program with PE teachers, this quantitative data will be used to investigate the qualitative data, by refining the questions asked of the qualitative data. This quantitative to qualitative approach to analysis will also occur vice versa, whereby initial codes of the qualitative data will be used to generate hypothesis and questions of the quantitative data.

Comparison and integration of findings from the different data sources will be guided by a triangulation protocol that seeks to interpret the findings using different methods to gain a more complete picture [40]. The triangulation protocol displays findings emerging from each component of the study on the same page. As such, a matrix will be produced to assess where findings from each method agree or partially agree (convergence), appear to contradict each other (discrepancy or dissonance) or are silent (i.e. where a theme or finding arises from one data set and not another, thus no complementarity) [40]. The aim of the triangulation protocol is to produce meta-themes that cut across the findings of individual methods [40]. An example of what this table could look like is in Table 3. The matrix will be used to highlight potential mechanisms that are essential for the implementation of PA4E1 physical activity practices.

**Table 3:** Example matrix: acceptability, appropriateness and feasibility of the PA4E1 implementation support strategies from the perspective of in-School Champions

Implementation support strategy:	Quantitative data	Quotes	Qualiative code	Convergence label (agree, slightly agree, silence, dissonance)
Strategy 1: *“Obtaining executive and leadership support”*				
Acceptability				
Appropriateness				
Feasibility				

## Discussion

This mixed methods process evaluation of PA4E1 is designed to investigate the implementation and mechanisms of impact of the program, with respect to context [16]. Findings will inform future improvement of PA4E1 and approaches to implement secondary school-based physical activity programs more broadly by comprehensively describing what was actually implemented and identifying elements of the implementation support strategy that may be particularly important in achieving beneficial effects. Whilst not commonplace, the publishing of process evaluation protocols clearly outlines key methodological choices, analysis methods and interpretation methods. This protocol contributes to only a small number of such process evaluation protocols, especially in physical activity, and with mixed methods designs [41]. Importantly, we acknowledge the importance of retaining flexibility to examine unforeseen hypothesis and events arising during the course of the program. The mixed methods design is suited to the potential for such exploration of unforeseen hypothesis and events [16].

### Strengths and Limitations

The prospective collection of a comprehensive set of implementation outcomes is a strength of this study, as well as the multi-disciplinary team and resources applied to this process evaluation. Whilst every effort will be made to clearly identify the student researcher responsible for the majority of qualitative data collection as part of the research team only, and not as part of the Implementation Team, if participants perceive the student researcher as aligned with program delivery (Implementation Team), this may have an impact on what participants disclose during interviews and focus groups. Despite this limitation to the PA4E1 process evaluation, the data will contribute to our understanding of the adoption or non-adoption of physical activity practices within schools. The outcomes of this process evaluation will contribute to the scarce amounts of implementation trial process evaluation data available for school-based physical activity programs.

## Trial Status

Protocol Version 1.0, 1 October 2019. Currently, the trial is ongoing and recruitment of participants for process evaluation continues. Recruitment began on 10 May 2017 and is expected to be completed by 20 December 2019.

## Supplementary information

**Additional file 1.** SPIRIT 2013 checklist.

**Additional file 2: Table 1**. Overview of the evidence based PA4E1 program (physical activity practices) including standards (essential and desirable) required of program schools [1].

**Additional file 3.** StaRI checklist. 

## Data Availability

Not applicable.

## Abbreviations

AIM: Acceptability of Intervention Measure
FIM: Feasibility of Intervention Measure
IAM: Intervention Appropriateness Measure
MRC: Medical Research Council
PA4E1: Physical Activity 4 Everyone
PE: Physical Education
RCT: Randomised controlled trial
SPIRIT: Standardized Protocol Items: Recommendations for Interventional Trials

## References

[1] Lee IM, Shiroma EJ, Lobelo F, Puska P, Blair SN, Katzmarzyk PT (2012). Effect of physical inactivity on major non-communicable diseases worldwide: An analysis of burden of disease and life expectancy. Lancet.

[2] Ding D, Lawson KD, Kolbe-Alexander TL, Finkelstein EA, Katzmarzyk PT, van Mechelen W, et al. The economic burden of physical inactivity: a global analysis of major non-communicable diseases. Lancet. 2016;388(10051):1311–24. Available from: 10.1016/S0140-6736(16)30383-X. 10.1016/S0140-6736(16)30383-X 27475266

[3] World Health Organization (2010). Global recommendations on physical activity for health.

[4] Hallal PC, Andersen LB, Bull FC, Guthold R, Haskell W, Ekelund U (2012). Global physical activity levels: surveillance progress, pitfalls, and prospects. Lancet.

[5] Telama R (2009). Tracking of physical activity from childhood to adulthood: a review. Obes Facts.

[6] World Health Organization. Global action plan on physical activity 2018–2030: more active people for a healthier world. 2018. Available from: https://www.who.int/ncds/prevention/physical-activity/gappa. Accessed 13 Sept 2019.

[7] Kriemler S, Meyer U, Martin E, van Sluijs EMF, Andersen LB, Martin BW. Effect of school-based interventions on physical activity and fitness in children and adolescents: a review of reviews and systematic update. Brit J Sports Med. 2011;45(11):923–30. Available from: 10.1136/bjsports-2011-090186. 10.1136/bjsports-2011-090186 PMC3841814 21836176

[8] Pate RR, Davis MG, Robinson TN, Stone EJ, McKenzie TL, Young JC. Promoting physical activity in children and youth: a leadership role for schools: a scientific statement from the American Heart Association Council on Nutrition, Physical Activity, and Metabolism (Physical Activity Committee) in collaboration with the Councils on Cardiovascular Disease in the Young and Cardiovascular Nursing. Circulation. 2006;114(11):1214–24. Available from: 10.1161/circulationaha.106.177052. 10.1161/CIRCULATIONAHA.106.177052 16908770

[9] Booth E, Halliday V, Cooper RJ. Headteachers’ and chairs of governors’ perspectives on adolescent obesity and its prevention in English secondary school settings. J Public Health. 2019; Available from: 10.1093/pubmed/fdz151. 31832667

[10] Nathan N, Elton B, Babic M, McCarthy N, Sutherland R, Presseau J, et al. Barriers and facilitators to the implementation of physical activity policies in schools: a systematic review. Prev Med. 2018;107:45–53 Available from: 10.1016/j.ypmed.2017.11.012. 29155228

[11] Love R, Adams J, van Sluijs EMF (2019). Are school-based physical activity interventions effective and equitable? A meta-analysis of cluster randomized controlled trials with accelerometer-assessed activity. Obes Rev.

[12] Borde R, Smith JJ, Sutherland R, Nathan N, Lubans DR (2017). Methodological considerations and impact of school-based interventions on objectively measured physical activity in adolescents: a systematic review and meta-analysis. Obes Rev.

[13] Naylor PJ, Nettlefold L, Race D, Hoy C, Ashe MC, Wharf Higgins J (2015). Implementation of school based physical activity interventions: a systematic review. Prev Med.

[14] Wolfenden L, Nathan NK, Sutherland R, Yoong SL, Hodder RK, Wyse RJ, et al. Strategies for enhancing the implementation of school-based policies or practices targeting risk factors for chronic disease. Cochrane Database Syst Rev. 2017;(11):CD011677 Available from: 10.1002/14651858.CD011677.pub2. PMC6486103 29185627

[15] Wolfenden L, Williams CM, Wiggers J, Nathan N, Yoong SL (2016). Improving the translation of health promotion interventions using effectiveness–implementation hybrid designs in program evaluations. Health Promot J Austr.

[16] Moore GF, Audrey S, Barker M, Bond L, Bonell C, Hardeman W (2015). Process evaluation of complex interventions: Medical Research Council guidance. BMJ.

[17] Lubans DR, Smith JJ, Skinner G, Morgan PJ. Development and Implementation of a smartphone application to promote physical activity and reduce screen-time in adolescent boys. Front Public Health. 2014;2:42. Available from: 10.3389/fpubh.2014.00042. 10.3389/fpubh.2014.00042 PMC4032995 24904909

[18] Norman Å, Nyberg G, Elinder LS, Berlin A (2016). One size does not fit all–qualitative process evaluation of the Healthy School Start parental support programme to prevent overweight and obesity among children in disadvantaged areas in Sweden. BMC Public Health.

[19] Sebire SJ, Edwards MJ, Kesten JM, May T, Banfield KJ, Bird EL (2016). Process evaluation of the Bristol girls dance project. BMC Public Health.

[20] Wilson DK, Griffin S, Saunders RP, Kitzman-Ulrich H, Meyers DC, Mansard L (2009). Using process evaluation for program improvement in dose, fidelity and reach: the ACT trial experience. Int J Behav Nutr Phys Act.

[21] Sutherland R, Campbell E, Nathan N, Wolfenden L, Lubans DR, Morgan PJ, et al. A cluster randomised trial of an intervention to increase the implementation of physical activity practices in secondary schools: study protocol for scaling up the Physical Activity 4 Everyone (PA4E1) program. BMC Public Health. 2019;19(1):883 Available from: 10.1186/s12889-019-6965-0. PMC6610944 31272421

[22] World Health Organization. What is a health promoting school? 2018 Accessed 18 April 2019; Available from: http://www.who.int/school_youth_health/gshi/hps/en/index.html.

[23] Bandura A (1986). Social foundations of thought and action: a social cognitive theory.

[24] Green LW, Richard L, Potvin L. Ecological foundations of health promotion. Am J Health Promot. 1996;10(4):270–81 Available from: 10.4278/0890-1171-10.4.270. 10159708

[25] Sutherland R, Campbell E, Lubans DR, Morgan PJ, Okely AD, Nathan N (2013). A cluster randomised trial of a school-based intervention to prevent decline in adolescent physical activity levels: study protocol for the ‘Physical Activity 4 Everyone’ trial. BMC Public Health.

[26] Sutherland RL, Campbell EM, Lubans DR, Morgan PJ, Nathan NK, Wolfenden L, et al. The Physical Activity 4 Everyone cluster randomized trial: 2-year outcomes of a school physical activity intervention among adolescents. Am J Prev Med. 2016;51(2):195–205. Available from: 10.1016/j.amepre.2016.02.020. 10.1016/j.amepre.2016.02.020 27103495

[27] Hollis JL, Sutherland R, Campbell L, Morgan PJ, Lubans DR, Nathan N (2016). Effects of a 'school-based' physical activity intervention on adiposity in adolescents from economically disadvantaged communities: secondary outcomes of the 'Physical Activity 4 Everyone' RCT. Int J Obes (Lond).

[28] Sutherland R, Reeves P, Campbell E, Lubans DR, Morgan PJ, Nathan N (2016). Cost effectiveness of a multi-component school-based physical activity intervention targeting adolescents: the ‘Physical Activity 4 Everyone’ cluster randomized trial. Int J Behav Nutr Phy Act.

[29] Standardized Protocol Items: Recommendations for Interventional Trials (SPIRIT) Accessed 13 February 2019; Available from: http://www.spirit-statement.org.

[30] Pinnock H, Barwick M, Carpenter CR, Eldridge S, Grandes G, Griffiths CJ (2017). Standards for Reporting Implementation Studies (StaRI) Statement. BMJ.

[31] Proctor E, Silmere H, Raghavan R, Hovmand P, Aarons G, Bunger A (2011). Outcomes for implementation research: conceptual distinctions, measurement challenges, and research agenda. Admin Pol Ment Health.

[32] Stirman SW, Miller CJ, Toder K, Calloway A (2013). Development of a framework and coding system for modifications and adaptations of evidence-based interventions. Implement Sci.

[33] Rabin BA, McCreight M, Battaglia C, Ayele R, Burke RE, Hess PL, et al. Systematic, Multimethod Assessment of Adaptations Across Four Diverse Health Systems Interventions. Frontiers in Public Health. 2018;6(102) Available from: 10.3389/fpubh.2018.00102. PMC5900443 29686983

[34] Wiltsey Stirman S, Baumann AA, Miller CJ (2019). The FRAME: an expanded framework for reporting adaptations and modifications to evidence-based interventions. Implement Sci.

[35] Weiner BJ, Lewis CC, Stanick C, Powell BJ, Dorsey CN, Clary AS (2017). Psychometric assessment of three newly developed implementation outcome measures. Implement Sci.

[36] Finch M, Stacey F, Jones J, Yoong SL, Grady A, Wolfenden L (2019). A randomised controlled trial of performance review and facilitated feedback to increase implementation of healthy eating and physical activity-promoting policies and practices in centre-based childcare. Implement Sci.

[37] QSR. NVivo qualitative data analysis Software - Version 12: QSR International Pty Ltd. 2018. Available from: http://www.qsrinternational.com/nvivo/support-overview/faqs/how-do-i-cite-nvivo-for-mac-nvivo-11-for-windows. Accessed 13 Sept 2019.

[38] Braun V, Clarke V. Using thematic analysis in psychology. Qual Res Psycol. 2006;3(2):77–101. 10.1191/1478088706qp063oa.

[39] Creswell JW, Clark VLP. Designing and conducting mixed methods research. 2nd ed. Los Angeles: SAGE Publications; 2011.

[40] O’Cathain A, Murphy E, Nicholl J (2010). Three techniques for integrating data in mixed methods studies. BMJ.

[41] Jong ST, Brown HE, Croxson CHD, Wilkinson P, Corder KL, van Sluijs EMF. GoActive: a protocol for the mixed methods process evaluation of a school-based physical activity promotion programme for 13–14year old adolescents. Trials. 2018;19(1):282. Available from: 10.1186/s13063-018-2661-0. 10.1186/s13063-018-2661-0 PMC5963130 29784016

