# Supplementary material for: Protocol for a mixed methods process evaluation of a hybrid implementation-effectiveness trial of a scaled-up whole-school physical activity program for adolescents: Physical Activity 4 Everyone (PA4E1)
Source: Trials. 2020 Mar 17;21:268. doi: 10.1186/s13063-020-4187-5 (PMC7077014; doi:10.1186/s13063-020-4187-5)
Supplement: Supplementary file 2 — Additional file 2: Table 1. Overview of the evidence based PA4E1 program (physical activity practices) including standards (essential and desirable) required of program schools [1]. [file 13063_2020_4187_MOESM2_ESM.docx]

**Additional File 2**

Additional File 2, Table 1. Overview of the evidence based PA4E1 program (physical activity practices) including standards (essential and desirable) required of program schools (1)

| ***Physical activity practices by Health Promoting Schools domain*** |
| --- |
| *Curriculum, teaching and learning* |
| 1. **Quality Physical Education (PE) lessons:**  - PE department (or equivalent PDHPE Department) used documented principles or guidelines for teachers to maximize PE quality, active learning time and student engagement in PE lessons (Program schools used the SAAFE principles- Supportive, Autonomous, Active, Fair, Enjoyable) (2). - Each PE teacher also participated in peer observation of a practical PE lesson, at least once a year*. - *Desirable – peer observation feedback is against the department’s quality PE principles.* |
| 1. **Student physical activity (PA) plans:**  - All Grade 7 students developed a personal PA plan which included  1. personal goals to improve or maintain activity or fitness 2. actions and timelines to achieve goals and 3. progress monitoring  - Goals reviewed once within year - *Desirable – students in Grade 7-10 develop a personal PA plan* |
| 1. **Enhanced school sport program:**  - The school delivered a short (10-12 weeks) structured PA Program designed to improve adolescents’ fitness and provide them with knowledge, motivation and skills to engage in a range of lifelong physical activities. - The program should be delivered to all students in at least one Grade between 7 and 10 (Program schools delivered the Resistance Training for Teens program to all of Grade 7 (3). |
| *Ethos and environment* |
| 1. **Recess/ lunchtime physical activity:**  - Supervised recess and/or lunchtime PA sessions offered to all students in Grades 7-10 at least 3 days per week. - PA equipment freely available to students at least three days per week at recess and/or lunch. - *Desirable - at least one organized recess or lunch activity per week targeting girls. Sessions promoted to students at least once per term.*      1. **School PA Policy or Procedure:**  - School developed a policy which included:  1. Provision of at least 150 minutes/week of MVPA during school time for all students in Grade 7-10; 2. Supportive practices to enhance all students’ PA (at least 3 of practices 1-4, 6-7 in this table)*** |
| *Partnerships and services* |
| 1. **Links with community physical activity providers:**  - At least three links that went beyond promotion of the provider (e.g. in newsletters) to involve an agreement, connection, partnership (e.g. out of hours sessions on school facilities, presentation by providers at school) were made. - Links were designed to support ‘outside of school time’ activity. - Links were communicated to students and families at least once per term****. - *Desirable - at least one of the community links made were to promote free or low cost options in the community.*  1. **Communicating physical activity messages to all parents**  - All parents of students in Grades 7-10 received PA messages that were designed to increase parent knowledge, attitudes and support towards PA, at least once per term. - These excluded messages only about school events e.g. carnivals, or school sports timetables or results, or promotion (advertisements for) community PA providers).**** |

*Program schools were asked to aim for peer observations once a semester

** Program schools asked to set plans for Grade 7 at 12 months, 7 and 8 at 24 months. For outcome assessment schools reporting plans developed for at least Grade 7 was considered sufficient

*** Program schools asked to include practices 1-4, 6 and 7 in their policy.

**** Program schools asked to use multiple modes to promote community links and to communicate PA information to parents (e.g. newsletters, parent app, parent information evening)

Additional File 2, Table 2. Overview of the multi-component implementation support intervention

| ***Implementation support strategies (n=7) and sub-strategies (n=23) (implemented over 4 school terms)*** |
| --- |
| **1. Executive and leadership support** |
| **1.1:** PA4E1 Partnership agreement signed by school executive. |
| **1.2:** New or existing school committee formed to oversee program. |
| **1.3:** The School committee is inclusive of in-School Champion and school executive to oversee the program. |
| **1.4:** Committee met at least once per term. |
| **2. Embedded school staff: in-School Champion** |
| **2.1:** An existing school PE teacher is allocated the role of in-School Champion to support implementation for full 12 months. |
| **2.2:** The position was funded by the NSW Department of Health, half day per week (equivalent to $350AUD a fortnight). |
| **3. External implementation support** |
| **3.1:** Health Promotion Support Officer (ideally a trained PE teacher) appointed to support schools with the program. |
| **3.2:** Health Promotion Support Officer was co-located within the relevant local health district. |
| **3.3:** Weekly contact was made with in-School Champion via phone, email and/or face-to-face site visits for 12 months. |
| **3.4:** Support Officer and in-School Champion have a face-to-face contact at least once a term. |
| **4. Teacher professional learning** |
| **4.1:** In-School Champion training –1-day of face to face training session was hosted by PA4E1 implementation team in Term 1. Accommodation, meals and transport costs were covered by the NSW Department of Health. |
| **4.2:** Quality PE training for all PE teachers - 6 x 10-minute online training videos followed by knowledge check short quizzes focused on the SAAFE principles were delivered via a password protected program website. |
| **4.3:** Enhanced school sport training – in-School Champions and other teachers involved in delivering the program could attend an existing 1 day face-to-face Resistance Training for Teens workshop offered by the NSW Department of Education (School Sport Unit), or equivalent training run by PA4E1 implementation team (not accredited). Course costs to be paid by project for in-School Champion, but not for other teachers. |
| **4.4:** School physical activity policy training – in-School Champion offered existing online training run by the NSW Department of Education School Sport Unit (Government schools only, n=19) (4). |
| **5. Resources** |
| **5.1:** Printed posters outlining Quality PE principles (SAAFE Principles (2)) to be displayed in PE department delivered to in-School Champions. |
| **5.2:** A $100AUD physical activity equipment voucher was provided to support the delivery of recess and lunchtime physical activity. |
| **5.3:** Equipment provided to support the delivery of recess and lunchtime physical activity enhanced schools sport program (5 Gymsticks/school) |
| **5.4:** Electronic resources housed on the program website (PA4E1 online) included:   - - overview of program presentation (Microsoft PowerPoint presentation)   - project milestones to be achieved each term (over 4 terms)   - online quality PE training (SAAFE Principle videos (6 videos - one overview and one per Principle) and worksheet, peer observation materials)   - student personal physical activity plan templates   - recess and lunch resources   - policy templates   - examples of community physical activity providers   - tips and frequently asked questions |
| **6. Provision of prompts and reminders** |
| **6.1:** Weekly emails or phone calls were made by the Support Officer to in-School Champions to encourage implementation. |
| **6.2:** Automated messages were sent each term via the program website to in-School Champions to prompt completion of teacher professional learning and online termly performance monitoring and feedback surveys. |
| **7. Implementation performance monitoring and feedback** |
| **7.1**: In-School Champion completes all termly surveys via the program website (PA4E1 Online). |
| **7.2**: A feedback report is automatically generated and sent to in-School Champions via email |
| **7.3**: A feedback report is automatically generated and sent to school Principals via email. |
|  |

Abbreviations: PE = Physical Education; PDHPE = Personal Development, Health and Physical Education; SAAFE = Supportive, Autonomous, Active, Fair, Enjoyable; PA = physical activity; MVPA = moderate-vigorous intensity physical activity)

References

1. Sutherland R, Campbell E, Nathan N, Wolfenden L, Lubans DR, Morgan PJ, et al. A cluster randomised trial of an intervention to increase the implementation of physical activity practices in secondary schools: study protocol for scaling up the Physical Activity 4 Everyone (PA4E1) program. BMC public health. 2019;19(1):883. Available via: <www.dx.doi.org/10.1186/s12889-019-6965-0>.

2. Lubans DR, Lonsdale C, Cohen K, Eather N, Beauchamp MR, Morgan PJ, et al. Framework for the design and delivery of organized physical activity sessions for children and adolescents: rationale and description of the ‘SAAFE’ teaching principles. International Journal of Behavioral Nutrition and Physical Activity. 2017;14(1):24. Available via: <www.dx.doi.org/10.1186/s12966-017-0479-x>.

3. Kennedy SG, Smith JJ, Morgan PJ, Peralta LR, Hilland TA, Eather N, et al. Implementing Resistance Training in Secondary Schools: A Cluster RCT. Med Sci Sports Exerc. 2017. Available via: <www.dx.doi.org/10.1249/mss.0000000000001410>.

4. NSW Department of Education. School Sports Unit - Developing procedures for school sport. 2019 [Accessed: 20/02/2019; Available via: <www.Gdurl.com/wEEE>
